# Calcified carotid artery atheromas on panoramic radiographs of head and neck cancer patients before and after radiotherapy

**DOI:** 10.4317/medoral.21436

**Published:** 2017-02-04

**Authors:** Renata-Lucena Markman, Karina-Gondim-Moutinho Conceição-Vasconcelos, Thais-Bianca Brandão, Ana-Carolina Prado-Ribeiro, Alan-Roger Santos-Silva, Márcio-Ajudarte Lopes

**Affiliations:** 1 DDS, MSc. DDS, PhD. Oral Diagnosis Department, Semiology Area, Piracicaba Dental School, University of Campinas (UNICAMP), Piracicaba, São Paulo, Brazil; 2 MD. Radiotherapy Service, Instituto do Câncer do Estado de São Paulo (ICESP), Faculdade de Medicina da Universidade de São Paulo, São Paulo, Brazil; 3 DDS, MSc. DDS, PhD. Dental Oncology Service, Instituto do Câncer do Estado de São Paulo (ICESP), Faculdade de Medicina da Universidade de São Paulo, São Paulo, Brazil

## Abstract

**Background:**

The aims of this study were to verify if head and neck radiotherapy (RT) is able to induce calcified carotid artery atheroma (CCAA) in a large head and neck cancer (HNC) population and also to compare the socio-demographic and clinical findings of patients with and without CCAA detected on panoramic radiographs.

**Material and Methods:**

Panoramic radiographs taken before and after head and neck radiotherapy (RT) of 180 HNC patients were selected and analyzed in order to identify the presence of CCAA. In addition, CCAA presence or absence on panoramic radiographs were compared and correlated with clinicopathological findings.

**Results:**

A high overall prevalence of CCAA was found on panoramic radiographs (63 out of 180 = 35%) of HNC patients. No significant difference of CCAA before and after RT was observed. There were also no differences between groups (with and without CCAA) regarding age, gender, tobacco and alcohol use, arterial hypertension, diabetes mellitus, acute myocardial infarction, hypercholesterolemia, tumor location, clinical stage of disease and RT dose. However, there was a greater prevalence of strokes in patients with CCAA (*p*<0.05).

**Conclusions:**

Although CCAA were frequently found in panoramic radiographs of patients with HNC, RT seems not to alter the prevalence of these calcifications.

**Key words:**Head and neck cancer, radiotherapy, carotid artery diseases, panoramic radiography.

## Introduction

HNC is the sixth most common cancer worldwide and approximately 686,000 new cases are diagnosed annually (1). The vast majority of these cases (95%) are squamous cell carcinoma (SCC) and most of them are in advanced clinical stages at diagnosis (2).

Therefore, treatment of these patients is very challenging and depends on the clinical stage, tumor site, patient’s age and medical condition. Nowadays the treatment is based on multi-modality approach and besides surgery, radiotherapy (RT) associated with chemotherapy is frequently used (3,4).

HNC patients commonly present with poor dental health at the moment of diagnosis and it is important that they could be evaluated by a dentist before RT (5). In this context, panoramic radiograph is helpful and more indicated in these patients, especially because of the pain or limited mouth opening (6).

Panoramic radiographs can also be useful for the identification of calcified carotid artery atheroma (CCAA), which appears as a round-shaped radiopaque image or two radiopaque vertical lines with a distance of 1.5 - 4.0 cm inferiorly to the angle of the mandible and/or between the posterior border of the mandible and the third and fourth cervical vertebrae (7,8).

Hyoid bone, triticeous cartilage, styloid process, superior horn of thyroid cartilage, epiglottis, calcified lymph nodes, tonsillolithiasis and salivary glands calcifications should be considered in the differential diagnosis of CCAA when identified in panoramic radiographs (7,8).

Head and neck RT is associated with acute and late toxicities and some studies suggested that RT may induce or accelerate atherosclerosis (9-12). Atherosclerosis is a chronic inflammatory disease which culminates in the formation of atheromatous plaques in the vasculature. Possible causes of endothelial dysfunction leading to atherosclerosis include elevated low-density lipoprotein cholesterol; free radicals caused by cigarette smoking, hypertension, and diabetes mellitus. Atheromas consist of a fibrous cap that overlies a core of lipid, necrotic tissue and calcium deposits within the arterial wall. Calcification of atheromatous lesions occurs due to the inflammatory process and is more frequent in vascular bifurcations as the carotid arteries. Clinical implications of calcified atheromas are coronary artery disease and ischemic stroke (13,14).

In an experimental study in mice, early-stage atherosclerosis was initiated one month after a single radiation dose of 14Gy. However, no increase in fatty streak formation was found 5 weeks after the start of fractionated irradiation (20 x 2Gy in 4 weeks) (15). Another study observed a significant increase in carotid intima media thickness (IMT) after RT of the neck and internal carotid arteries (ICA) of irradiated patients. However, in the group of unilateral irradiated patients where IMT of CCAA and ICA were compared between the irradiated and the non-irradiated sides of the same patient, no statistically significant increases were noticed (16). In another study, Freymiller *et al.* (9) used panoramic radiograph to assess carotid artery atherosclerotic development. They found that approximately 53% of the patients developed a calcified atheroma 69.7 months after conclusion of radiation therapy. Nevertheless, their research had a small study population of 17 patients.

As no consensus has been achieved, the aim of this study was to verify if head and neck RT is able to induce CCAA in a large HNC population and also to compare the socio-demographic and clinical findings of patients with and without CCAA detected on panoramic radiographs.

## Material and Methods

- Patients’ selection and data retrieval

This research was approved by the Ethical Committee of the Faculdade de Medicina da Universidade de Sao Paulo, Brazil protocol number 882.731. A retrospective design consisted of 180 patients with head and neck SCC with the carotid arteries included in the radiation field treated with 3D conformal RT in 6mV linear accelerators on Synergy Platform (Elekta AB, Stockholm, Sweden) from January 2012 to August 2014. All patients included in the study had panoramic radiographs taken before and after the conclusion of head and neck RT. The panoramic radiographs were taken to access dental health of the patients at all times.

Socio-demographic variables, comorbid medical illnesses, clinical stage of cancer at diagnosis, tumor location, tobacco and alcohol use and treatment information were collected from the patient’s medical records.

- Panoramic radiograph evaluation

The patient’s panoramic radiographs were all digital and taken in the same dental X-ray machine (PaX-400, Hawseong-si, Gyeonggi-do, Korea), using 68KV*p*, 8 mA with an exposure time of 14s maintaining the quality and standardization of the images.

Radiographs were coded with a specific tag so the name and other clinical data of patients were preserved. Radiographic images were evaluated for the presence of CCAA by two certified oral medicine practitioners reaching substantial agreement of 0.675 on Kappa test. A third opinion was sought in case of disagreements. Images were displayed on a 13.3 inch LCD laptop (Vostro 1320, Dell Inc., USA) with a screen resolution of 1280 x 800 pixels. All assessments were done in the same viewing room with optimal lighting and viewing conditions.

The panoramic radiographs were evaluated for the followings signs: a round-shaped radiopaque image or two radiopaque vertical lines with a distance of 1.5 - 4.0 cm inferiorly to the angle of the mandible and/or between the posterior border of the mandible and the third and fourth cervical vertebrae according to Friedlander in 1995 (7).

After examining the radiographs, patients were divided into 2 groups: without CCAA (group 1) and with CCAA (group 2) and had their clinical information compared.

- Definition of risk factors

Arterial hypertension was defined as blood pressure >140/90 mmHg and/or use of blood pressure reducing medication; hypercholesterolemia was defined as ongoing use of lipid-lowering medication and diabetes mellitus was defined as ongoing use of oral hypoglycemic medication.

- Statistical analyses

Descriptive statistics (frequency, percentage) and comparisons of patient’s characteristics between those with and without radiographically evident CCAA were calculated using Chi-squared test and Likelihood ratio test when the expected numbers in cells were less than 5. The α level for each statistical comparison was set at 0.05.

## Results

The study population consisted of 180 patients and the vast majority was male (83.89%) with a mean age of 59.4 years (ranging from 20 to 85 years), being most of them (70.56%) between 50 and 70 years. Regarding the habits, most of the patients were smokers and/or alcohol drinkers.

Oropharynx (33.89%), oral cavity (26.67%) and larynx (25%) were the most frequent tumor locations and 162 patients (90%) were diagnosed in clinically advanced stages III and IV. All patients were submitted to 3D - conformal exclusive or adjuvant RT (mean dose of 65.73 Gy, ranging from 30 to 70Gy) and most of them (76.67%) received a total dose between 60 and 70 Gy. Chemotherapy was used in 136 patients (75.56%) and surgery was performed in 78 patients (43.33%).

A review of their medical histories showed that 63 patients (35%) had arterial hypertension, 22 (12.22%) were diabetic, 11 (6.11%) were in use of lipid-lowering medication, 5 (2.78%) suffered a previous stroke and 5 (2.78%) had a history of acute myocardial infarction.

Of the 180 patients enrolled, 57 (31.67%) were identified with CCAA in both radiographs (before and after RT), 4 (2.22%) presented CCAA only in radiographs after RT and 2 (1.11%) only in radiographs before RT. None of the patients with CCAA in both radiographs (57, 31.67%) showed an increase in size or different morphology of the CCAA after RT.

Differences related to gender, age, tobacco and alcohol use, tumor location and clinical stage, RT dose, comorbidities as arterial hypertension, diabetes mellitus, hypercholesterolemia and acute myocardial infarction were not significant between those with and those without CCAA ( Table 1).

**Table 1:**
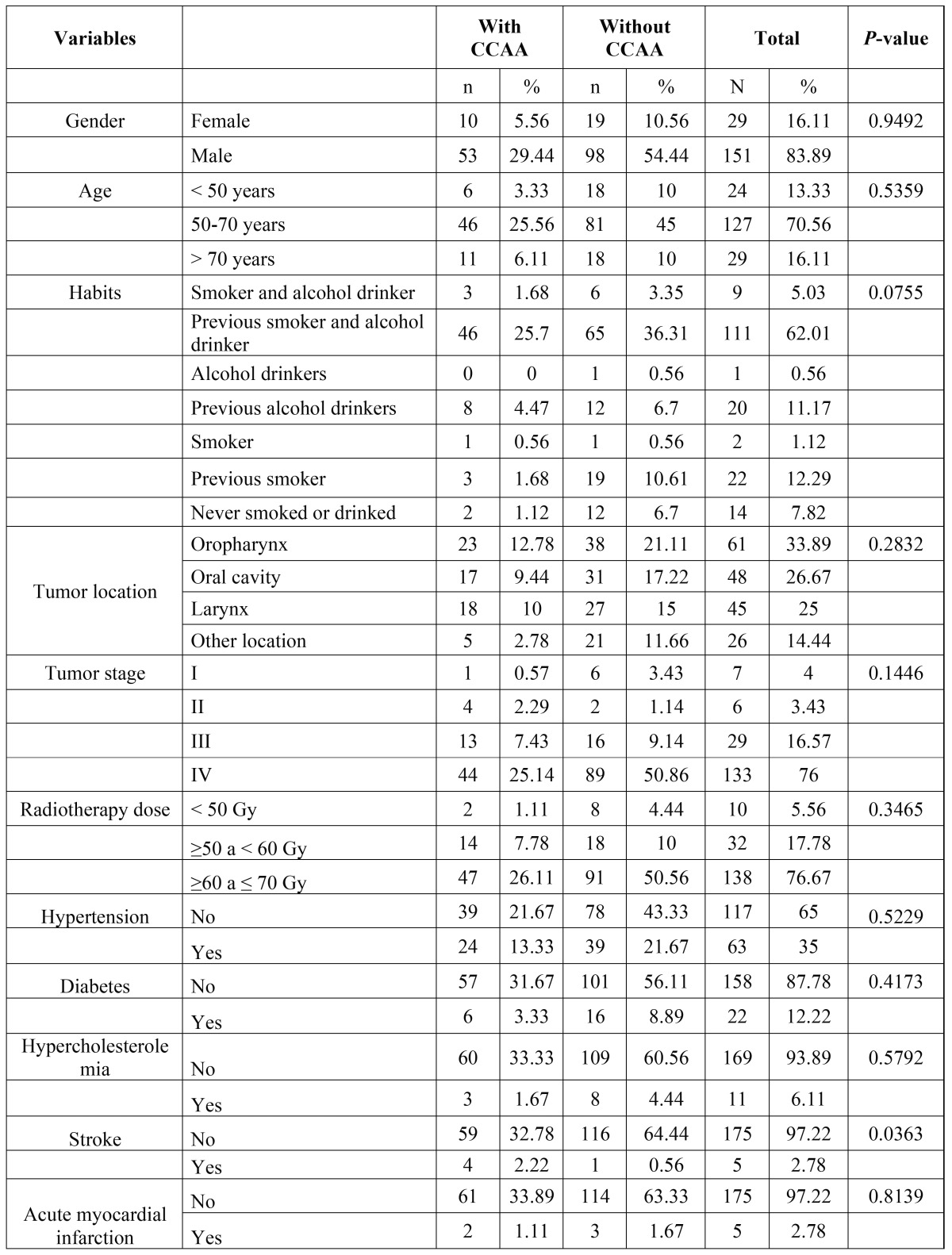
Comparison of socio-demographic findings and comorbidities of patients with and without CCAA.

However, 4 out of 5 patients with previous history of stroke were identified with CCAA on their panoramic radiograph (*p*<0.05). Patients with CCAA were 7.42 (CI 95% 6,95-7,94) times more predisposed to cerebrovascular events then patients without CCAA.

The mean time between the radiographs before and after RT was of 9.06 ± 6.70 months.

## Discussion

The present study reports a retrospective analysis about the role of RT in inducing CCAA in HNC patients evaluated by panoramic radiograph. It has been described that these patients present a greater prevalence of CCAA when compared to the general population (17). The current paper presented a prevalence of 35% CCAA on panoramic radiograph in HNC population. This finding was inferior of Friedlander’s *et al.* (18) (2012), who observed that 52% of individuals diagnosed with HNC before undergoing RT had these calcifications. The difference in the prevalence rates between the 2 studies most likely occurred because of the age of the patients, dietary habits, tobacco and alcohol consumption that vary among distinct geographic areas. In 2010, Strenja-Linič *et al.* (19) conducted a research to evaluate the prevalence of carotid artery stenosis in irradiated patients for larynx carcinoma using ultrasonography. Their results were very similar to the current study, where 33.33% of their population presented a significant carotid artery stenosis and in most patients an atheromatous plaque was also found.

Many studies have suggested that the direct effect of RT to the carotid arteries is able to induce/accelerate atherosclerosis (16,20-22). In the current research, most of the patients with CCAA in their panoramic radiograph after RT also presented this alteration before treatment. Therefore, these findings suggest that RT was not able to change the prevalence of CCAA in this population. Though it is important to highlight that this research had a limited time of follow-up.

The patients in this study were predominantly diagnosed in clinical advanced stages (76.11%) and received high doses of chemo and radiotherapy. Therefore it was estimated that this population would have a high prevalence of CCAA. This didn’t prove true because even though it had a higher prevalence than the general population, it was not higher than some studies (18,19).

Patients with systemic diseases such as diabetes mellitus, chronic renal failure, which are commonly associated with arterial hypertension and coronary disease, and long-time smokers present more CCAA than the general population (2.76 - 4.8%) (23-25). Therefore, risk factors for atherosclerosis, such as hypercholesterolemia, arterial hypertension, diabetes mellitus, smoking and increasing age that are frequently found in HNC patients may represent an important bias in studies that consider RT capable of inducing CCAA.

The clinicopathological and demographic findings of these patients were consistent with those of most of the studies on HNC, where patients are usually men in the sixth or seventh decades of life and are diagnosed in advanced clinical stages (26,27).

In this research, no significant differences in comorbidities were observed between patients with and without CCAA. These findings are similar to those of Friedlander *et al. * (2012) (18). Nevertheless, in their studied population the presence of hypertension was significantly greater in the group with atheromas (60% vs. 30.4%) and all patients were evaluated before undergoing RT. Toprak *et al.* (2012) also found that the presence of risk factors did not influence significantly IMT of carotid arteries after RT (20).

The identification of carotid atheroma in panoramic radiographs represents an important predictive feature. Individuals with CCAA on their panoramic radiographs have an increased risk of future vascular events when compared to age and vascular risk factors matched controls (28,29). In this research, 80% of the patients with a previous stroke were identified with CCAA on their panoramic radiographs.

This retrospective study conducted with HNC patients panoramic radiographs sought to assess if RT to the head and neck could induce the formation of CCAA. However, cross-sectional studies present clear limitations regarding the time of follow-up and temporal relationship of the facts. Notwithstanding, even though panoramic radiographs are not the most indicated exams to evaluate the presence of CCAA, they are extremely useful for early detection in asymptomatic patients (25). Furthermore, this research had a greater number of patients (180) than the majority of published articles (10,16) and all panoramic radiographs were digital, which has a superior quality when compared to conventional ones (9,18,22). Moreover, all patients were submitted to 3D conformal RT for treatment of a SCC of the head and neck with carotid arteries included in the radiation field - making it a more homogeneous group.

In conclusion, RT did not alter the prevalence of CCAA on panoramic radiographs of patients with HNC during this time of follow-up, but these calcifications are frequently found in this population and are associated with a greater risk of developing a stroke. Therefore, it is important that dentists be aware of the presence of CCAA in panoramic radiographs when caring for patients with a history of irradiation to the head and neck area.
